# Biallelic *FGF4* Variants Linked to Thoracic Dystrophy and Respiratory Insufficiency

**DOI:** 10.1111/cge.14758

**Published:** 2025-04-22

**Authors:** Laura M. Watts, Esther Kinning, Donald R. Latner, Marla Johnston, Jessica Patrick‐Esteve, Gregory M. Cooper, Stephen R. F. Twigg, Alistair T. Pagnamenta, Jenny C. Taylor

**Affiliations:** ^1^ Oxford NIHR Biomedical Research Centre, Centre for Human Genetics University of Oxford Oxford UK; ^2^ Oxford Centre for Genomic Medicine Nuffield Orthopaedic Centre Oxford UK; ^3^ West Midlands Regional Genetics Service Birmingham Women's Hospital Birmingham UK; ^4^ HudsonAlpha Institute for Biotechnology Huntsville Alabama USA; ^5^ Children's Hospital New Orleans New Orleans Louisiana USA; ^6^ Department of Pediatrics Louisiana State University Health Sciences Center New Orleans Louisiana USA; ^7^ Clinical Genetics Group, MRC Weatherall Institute of Molecular Medicine University of Oxford Oxford UK

**Keywords:** fibroblast growth factor 4, pulmonary hypoplasia, skeletal dysplasia, thoracic dystrophy

## Abstract

The thoracic dystrophies are inherited skeletal conditions where abnormal embryonic development of the thoracic skeleton results in a narrow chest, pulmonary hypoplasia, and respiratory insufficiency, which can be severe or lethal. The majority of thoracic dystrophies are due to biallelic alterations in genes needed for normal ciliary function. However, despite the identification of over 20 genes as causal for the thoracic dystrophy phenotype, around 20% of patients remain without a molecular diagnosis. We present two unrelated families with a clinical diagnosis of thoracic dystrophy with associated respiratory insufficiency without a molecular diagnosis on previous genetic testing. Both harbor rare biallelic and predicted deleterious missense substitutions in *FGF4*, a gene known to be essential for formation of the thoracic skeleton in mice. We demonstrate that the phenotype is restricted to short ribs, abnormally narrow chest, and respiratory insufficiency, without other diagnostic clinical or radiological signs. We suggest that biallelic alterations in *FGF4* are a newly identified disease association of thoracic dystrophy.

## Graphical abstract

We report three people from two unrelated families with a clinical diagnosis of thoracic dystrophy characterized by short or missing ribs, narrow chest, and respiratory insufficiency. Affected people each have rare, biallelic, predicted deleterious missense substitutions in *FGF4*, a gene known to be essential for the formation of the thoracic skeleton in mice.

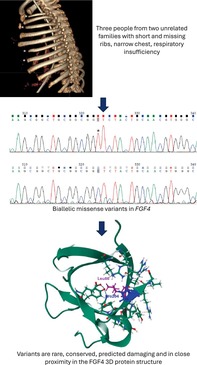

## 1 Introduction

The thoracic dystrophies are a group of typically autosomal recessively‐inherited, skeletal conditions where short ribs and a narrow chest result in pulmonary hypoplasia [1, 2]. The spectrum of disease ranges from in utero or neonatal death to survival into adulthood [1]. Many of the recognized genetic causes of thoracic dystrophy involve genes essential for the function of cilia, highly specialized cellular organelles with key functions in human development [3]. While many ciliopathies have multi‐organ manifestations commonly affecting the kidney, brain, and eyes, a subset referred to as the skeletal ciliopathies are characterized by thoracic dystrophy, with or without other organ system manifestations [1]. Recognized skeletal ciliopathy syndromes include the short rib polydactyly and Jeune syndromes (*DYNC2H1* and components of the ciliary intraflagellar transport pathway, among others), and Ellis‐van Creveld syndrome (*EVC1* and *EVC2*) [1, 2].

With dedicated analysis, a genetic diagnosis can be made in the majority of patients with a thoracic dystrophy [3]. However, there remains a proportion of patients with a clinical diagnosis of thoracic dystrophy without a corresponding molecular diagnosis [1], likely representing variants in established genes that have evaded detection or novel disease‐causing genes. Novel causes of thoracic dystrophy may include genes encoding components of the ciliary apparatus not yet associated with human disease, or variants affecting other key genes and pathways involved in the formation of the rib cage.

Fibroblast growth factors (FGF) and their four corresponding tyrosine kinase fibroblast growth factor receptors (FGFR) are widely expressed in the skeleton and are essential for axial and appendicular skeletal development [4, 5, 6, 7]. Altered FGF signaling causes multiple human monogenic skeletal diseases [2, 4]. FGFs, including FGF4 and FGF8, are needed for vertebral and rib cage development [5, 6], while FGFR signaling also affects primary cilium length, including in cartilaginous growth plates and chondrocytes [8, 9]. We report two families with a clinical diagnosis of thoracic dystrophy without a molecular diagnosis. Whole genome sequencing (WGS) identified homozygous variants in *FGF4*, which have not been previously associated with any human disease phenotype.

## 2 Materials and Methods

Family 1 was recruited to the UK 100,000 Genomes Project (100KGP) for WGS under the disease category of thoracic dystrophy [10]. Family 2 was recruited for WGS through SouthSeq, a clinical research study that enrolled infants with suspected genetic disorders as part of the Clinical Sequencing Evidence‐Generating Research consortium (CSER) [11]. Variants were assessed for likely pathogenicity using in silico variant prediction tools, population allele frequencies, protein conservation, and in silico structural analysis (Supporting Information).

## 3 Results

Detailed case reports are available in Supporting Information.

### 3.1 Family 1

**FIGURE 1:**
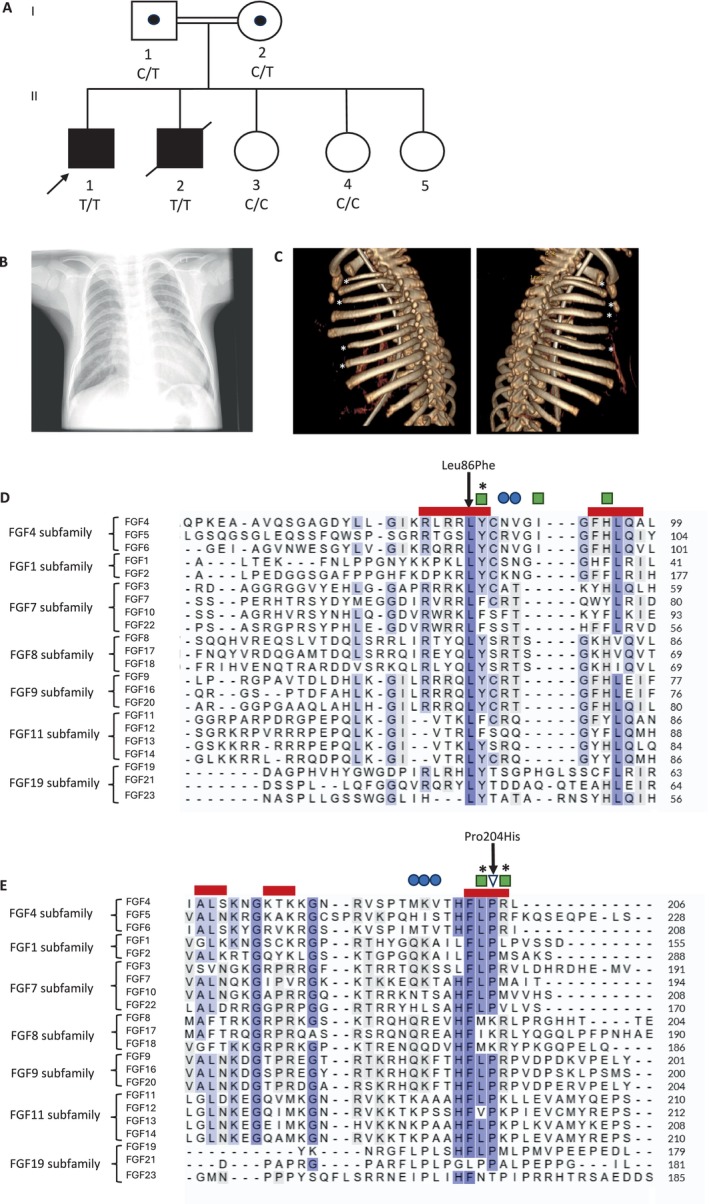
Pedigree, radiological images and protein sequence alignments of reported FGF4 variants. (A) Pedigree of Family 1. Colored black boxes represent individuals with a clinical diagnosis of a short rib thoracic dystrophy. Both affected children were homozygous for a missense variant in *FGF4* (c.256C>T p.(Leu86Phe)). Both parents are confirmed carriers. Two of three unaffected siblings do not carry the variant. Genotypes are indicated for individuals as C (reference allele) and T (missense variant). (B) Chest x‐ray of II‐1 from Family 1 aged 3 years 6 months showing a small thorax with short ribs. Only 11 pairs of ribs are present. (C) 3D reconstruction of CT scan of II‐1 from Family 2 demonstrating hypoplasia of the 1st, 3rd, 6th and 7th ribs on the left and 1st, 3rd, 4th and 6th ribs on the right (marked with white stars). (D and E) Sequence alignment around Leu86 (D) and Pro204 (E). Amino acid sequences of all human FGFs were retrieved from Uniprot and aligned using the Clustal Omega algorithm, grouped by FGF subfamily [12]. Positions of the missense FGF4 alterations are indicated by arrows. Red boxes represent β‐strands of the FGF domain in FGF4; blue circles represent heparin binding sites; triangle represents a residue interacting with the D2–D3 linker region; residues that contact the D2 immunoglobulin domain of FGFR1 are shown by green squares; asterisks denote residues which, when altered, have reduced receptor binding and FGF activity [12, 13].

Family 1 (Table 1) comprises two affected boys clinically suspected to have Jeune syndrome, and three unaffected children born to healthy consanguineous 2nd cousin parents (Figure 1A). The proband (II.1) was born at term following an uncomplicated pregnancy by normal vaginal delivery. At birth, he required resuscitation for respiratory distress and was ventilated for the first 6 months of life. He had a very small thorax on clinical examination and was diagnosed with pulmonary hypoplasia. He required non‐invasive ventilation with overnight ventilatory support until 20 months old. Development was normal, and he is now 20 years old.

**TABLE 1:** Summary of clinical characteristics of affected individuals from two families.

Family	Individual	Sex	Age at assessment	Antenatal findings	Neonatal resuscitation	Chest size and shape	Ribs	Spine abnormalities	Limb abnormalities	Respiratory insufficiency	Echo	Other
1	II.1	M	20 years	No	Yes.	Narrow thorax	Short ribs, 11 pairs	No	No	Ventilated for first 6 months of life, then non‐invasive ventilation. Overnight ventilatory support until 20 months	Normal	Pyloric stenosis
	II.2	M	Deceased 3 months from respiratory infection		Yes	Small thorax	Short ribs, 11 pairs	No	No	Chronic respiratory insufficiency	Normal	NA
2	II.1	M	2 years	Small bell shaped throat, short ribs, lung volume small for gestational age	No	Thoracic dysplasia	Short ribs with hypoplasia	Secondary lumbar scoliosis	No	Pulmonary hypoplasia and chronic respiratory insufficiency, dependent upon long term mechanical ventilation via tracheostomy	PFO	Gastrostomy

Abbreviations: M, Male; PFO, patent foramen ovale.

His younger brother (II.2) was born at term by vaginal delivery. At 1 h old, he was ventilated for respiratory distress. He was mildly hypotonic and had a small thorax with chronic respiratory insufficiency. He passed away at 3 months old following a respiratory infection.

X‐rays demonstrated a small thorax, short ribs, and only 11 pairs of ribs in both brothers (Figure 1B). The proband was recruited to 100KGP together with his unaffected parents and two unaffected siblings. Primary analysis using the standard 100KGP pipeline did not identify any causative variants in relevant disease panels [10].

### 3.2 Family 2

The proband (Table 1) is a male child aged two at last assessment, born at 35 + 1 weeks to non‐consanguineous parents. There was no relevant family history. The pregnancy was complicated by maternal methadone use, smoking, and trichomonas infection. Antenatal ultrasound showed a small thorax suspicious of a skeletal dysplasia. Fetal MRI confirmed a small bell‐shaped thorax with short ribs and low lung volume for gestational age (total 30.3 mL, < 2.5% expected, right lung 15.7 mL, left lung 14.6 mL). No prenatal genetic testing was performed.

Postnatally, his chest shape was in keeping with thoracic dystrophy and a clinical suspicion of Jeune syndrome. Chest CT demonstrated shortening and hypoplasia of the 1st, 3rd, 4th and 6th ribs on the right and 1st, 3rd, 6th and 7th ribs on the left, with otherwise normal lungs (Figure 1C). An echocardiogram demonstrated a patent foramen ovale with left‐to‐right shunting, with normal left ventricular size and systolic function. Aged two, he is dependent upon long‐term mechanical ventilation via a tracheostomy for pulmonary hypoplasia. He has impairments in speech, language, and oral motor skills, is fed via a gastrostomy, and has lumbar scoliosis. Chromosomal microarray was normal. No pathogenic variants were identified on a skeletal ciliopathies gene panel, only a heterozygous variant of uncertain significance in the recessive gene *WDR60*.

### 3.3 Genetic Analysis

All three affected individuals from the two unrelated families harbored homozygous missense variants in *FGF4*. In Family 1, further research analysis of musculoskeletal cases recruited to 100KGP (Supporting Information) identified a homozygous missense variant in *FGF4* NM_002007.4:c.256C>T p.(Leu86Phe), absent from two unaffected siblings and present in a heterozygous state in both parents. Sanger sequencing on stored DNA from the deceased similarly affected sibling demonstrated the same homozygous *FGF4* missense variant (S1A). Research WGS in Family 2 demonstrated a biallelic missense variant in *FGF4* NM_002007.4:c.611C>A p.(Pro204His). Parents were both heterozygous carriers.

Both missense variants are considered to be deleterious by multiple in silico tools, with CADD scores of > 25 and annotated by AlphaMissense as likely pathogenic (Table 2) and are absent from the gnomAD database [14]. Present in gnomAD are two individuals with a different missense substitution at the same position as the Family 1 variant (p.(Leu86Pro)) in the heterozygous state where no phenotype would be expected, and 24 individuals homozygous for common polymorphisms in *FGF4*, which, in contrast to our variants, have little evidence for pathogenicity (Supporting Information).

Leu86 and Pro204 are both highly conserved. At position 86, there is a Leucine in all 22 human FGFs (Figure 1D) and in multiple species, including invertebrates (S1B). At position 204, there is a proline in the majority of human FGFs (Figure 1E) and vertebrates, but with greater diversity in invertebrates, where there are fewer paralogs (S1C).

Both *FGF4* missense alterations are found in β strands of the core FGF domain responsible for binding to the FGF receptor [13], and are brought into close proximity in the 1.80 Å 3D protein structure (S1D). Leu86 lies immediately adjacent to Tyr87, which contacts the D2 immunoglobulin‐like domain of FGFR1 and which, when altered, results in reduced receptor binding and FGF activity [13] (Figure 1D). Leucine at position 86 is a hydrophobic amino acid buried in the core of the FGF4 protein. Alteration to the bulkier phenylalanine is likely to disrupt the conformation and interactions of FGF4 (S2), supported by in silico modeling predictions of altered hydrogen bonding involving highly conserved FGFR‐contacting residues [13] (S2).

Proline 204 directly interacts with the D2‐D3 linker region of FGFR1 [13], which contributes to ligand binding and specificity [13, 15]. Therefore, substitution to histidine would be consistent with loss of function. Specific missense substitutions in this linker region in FGFR1, FGFR2, and FGFR3 cause Pfeiffer, Apert, and Muenke syndromes, respectively [16]. Pro204 is additionally flanked by two residues that contact the D2 domain of FGFR1, and alteration of which results in reduced receptor activity [13].

**TABLE 2:** Homozygous *FGF4* variants reported in this study (transcript NM_002007.4).

Family	Individual	Parental consanguinity	Ethnicity	Homozygous *FGF4* variant	Exon, domain	gnomAD/UKBB frequency	CADD	AlphaMissense	SIFT	PolyPhen	REVEL
1	II.1	Yes	Asian or British Asian: Indian	c.256C>T p.(Leu86Phe)	1, beta strand of FGF domain	0/0	26.1	0.95 (likely pathogenic)	0 (deleterious)	1 (probably damaging)	0.78
	II.2			c.256C>T p.(Leu86Phe)							
2	II.1	No	European American	c.611C>A p.(Pro204His)	3, beta strand of FGF domain	0/0	29.5	0.99 (likely pathogenic)	0 (deleterious)	0.99 (probably damaging)	0.816

*Note:* Domains from DECIPHER and SMART, allele frequency from gnomAD v.4.1.0, annotation from Ensembl Variant Effect Predictor and REVEL (https://sites.google.com/site/revelgenomics). UK Biobank information was accessed at https://afb.ukbiobank.ac.uk/gene/FGF4.

## 4 Discussion

We describe three individuals from two families with thoracic dystrophy and pulmonary hypoplasia, each with homozygous missense variants in *FGF4* in the receptor‐binding domain. The variants are absent from gnomAD and UK Biobank [14], are predicted to be damaging by multiple in silico tools, and are immediately adjacent to receptor‐contacting residues required for normal FGF signaling [13]. Consistent with a loss‐of‐function mechanism through altered binding to FGF receptors, Pro204 has been shown to directly contact FGFR1 [13], while the extremely highly conserved Leu86 residue is mutated to a bulkier amino acid likely to compromise structural conformation.

The vertebrae and rib cage form from somites, which periodically segment from the presomitic mesoderm in a process requiring FGF4 [5, 17]. Mice lacking *Fgf4* alone or in combination with *Fgf8* demonstrate a spectrum of vertebral and rib defects resulting from abnormal somitogenesis with similarities to the phenotypes described in our patients, including missing ribs and an abnormally shaped thoracic cage [6, 17]. In mice, inactivation of *Fgf4* in the presomitic mesoderm has also been shown to result in abnormal patterns of expression of both *Mesp2* and *Hes7* [17]. Human pathogenic alterations in *MESP2* and *HES7* cause spondylocostal dysostosis characterized by rib and vertebral abnormalities [18]. Nevertheless, despite the important role of FGF4 in somitogenesis, formal confirmation of the pathogenicity of the described variants requires dedicated functional studies such as study of variant‐specific model organisms, in vitro assessment of mutant FGF4‐receptor binding, and investigation of ciliary signaling pathways. Identification of additional affected families would further support this work.

Many ciliopathies have multiple organ manifestations resulting from diverse ciliary functions during development [1, 3]. In contrast, the families reported had a phenotype largely restricted to thoracic dystrophy. Although FGF receptor signaling has been shown to be involved in the regulation of cilium length [8, 9], and in zebrafish *Fgf4* may be needed for normal cilia development [19], *FGF4* is not well established as an essential ciliary gene in humans or mice. The phenotype reported does not include limb shortening or characteristic radiological features such as trident acetabulum found in other thoracic dystrophies [1], nor characteristic clinical or radiological features of Kagami‐Ogata syndrome. In mice, *Fgf4* is known to be expressed in the apical ectodermal ridge responsible for normal limb bud outgrowth [7, 20], functional redundancy with *Fgf8* means that loss of *Fgf4* alone does not result in a limb phenotype [7, 20].

Clinically, we have been able to provide a likely diagnosis for the deceased sibling in Family 1, and reproductive counseling for his surviving 20‐year‐old brother. Identification of novel rare disease genes allows expansion of genetic testing panels and biological insights which may aid research into specific treatments. Our work suggests that homozygous variants in *FGF4* may lead to a phenotype of thoracic abnormalities with respiratory insufficiency, which could prompt screening of other rare disease cohorts, or inclusion in diagnostic testing panels.

## Author Contributions

**Laura M. Watts, Alistair T. Pagnamenta, Jenny C. Taylor:** study conception. All authors: data acquisition. **Laura M. Watts:** manuscript initial drafting. All authors: manuscript revision, editing, and approval.

## Ethics Statement

Ethics approval for the 100,000 Genomes Project was from Cambridge South REC (14/EE/1112). The review board at the University of Alabama at Birmingham (IRB‐300000328) approved and monitored the SouthSeq study (family 2).

## Consent

All patients or their legal guardians gave signed informed consent for publication.

## Conflicts of Interest

The authors declare no conflicts of interest.

## Supporting information


**Data S1.** Supporting Information.

**Data S2.** Supporting Information.

**Data S3.** Supporting Information.

## Data Availability

The data that support the findings of this study are available from the corresponding author upon reasonable request.
